# Importance of Examined Lymph Node Number in Accurate Staging and Enhanced Survival in Resected Gastric Adenocarcinoma—The More, the Better? A Cohort Study of 8,696 Cases From the US and China, 2010–2016

**DOI:** 10.3389/fonc.2020.539030

**Published:** 2021-01-06

**Authors:** Lei Huang, Xinyue Zhang, Zhijian Wei, Aman Xu

**Affiliations:** ^1^Department of General Surgery, the First Affiliated Hospital of Anhui Medical University, Hefei, China; ^2^Department of Academic Research, Hefei City First People’s Hospital, Hefei, China

**Keywords:** gastric adenocarcinoma, examined lymph node count, accurate staging, stage migration, long-term survival, multivariable breakpoint analysis, large cohort study

## Abstract

**Background:**

While most guidelines advocate D2 lymphadenectomy for non-metastatic gastric adenocarcinoma (nmGaC), it is not always performed as standard of care outside East Asia. The recommended minimal examined lymph node (ELN) count in nmGaC to stage cancer accurately varies largely across guidelines, and the optimal count to satisfactorily stratify patient survival has yet to be determined. This large cohort study aimed at robustly defining the minimal and optimal thresholds of examined lymph node (ELN) number in non-metastatic gastric adenocarcinoma (nmGaC).

**Methods:**

Data on nmGaC patients operated in 2010–2016 and surviving ≥3 months were retrieved from the US SEER-18 Program and a Chinese multi-institutional gastric cancer database (MIGC). The correlation of ELN count with stage migration and patient survival were quantified with the use of the multivariable-adjusted logistic and proportional hazards Cox models, respectively. The sequences of odds ratios (ORs) and hazard ratios (HRs) for each additional ELN were smoothed, and the structural breakpoints were determined.

**Results:**

Together 7,228 patients from the US and 1,468 from China were analyzed, encompassing 23,114 person-years of follow-up. The mean ELN count was 20 in the US and 30 in China. With more ELNs, both cohorts significantly showed proportional increases from lower to higher nodal stage (OR_SEER_ = 1.03, 95%-CI = 1.03–1.04; OR_MIGC_ = 1.02, 95%-CI = 1.02–1.03) and sequential enhancements in postoperative survival (HR_SEER_ = 0.97, 95%-CI = 0.97–0.97; HR_MIGC_ = 0.98, 95%-CI = 0.97–0.99). Correlations for both stage migration and survival were still significant in most subgroups by patient, cancer, and management factors. Breakpoint analyses revealed a minimum threshold ELN count of 17 and an optimum count of 33, which were validated in both cohorts with good efficacy to differentiate probabilities of both stage migration and survival.

**Conclusion:**

In resected nmGaC patients with anticipated survival ≥3 months, more ELNs are correlated with more accurate staging, which may partly explain the survival correlation. This observational investigation does not indicate causality. Our findings robustly conclude 17 ELNs as the minimum and propose 33 ELNs as the optimum thresholds, to assess the quality of lymph node examination and to stratify postsurgical survival.

## Background

Gastric cancer, the majority of which is adenocarcinoma, ranks fifth in cancer incidence and is the third leading cause of cancer-related fatality globally, with more than 1,000,000 new cases and about 783,000 deaths in 2018 (1). Resection with adequate lymphadenectomy is still the cornerstone of potential cure for most medically fit patients with non-metastatic gastric adenocarcinoma (nmGaC) (2). While most guidelines (3–15) advocate D2 lymphadenectomy in specialized centers with experienced surgeons, it is not always performed as standard of care outside East Asia.

Lymph node (LN) status is a key prognosis predictor and an important determinant for postsurgical treatment decision-making in patients with nmGaC (16). Adequate LN removal and examination may contribute to enhanced treatment outcomes by clearing possible metastatic nodes and to accurate nodal staging by affecting stage migration (17–19). Accurate staging is the prerequisite for adequate administration of adjuvant therapy. For instance, while adjuvant chemotherapy is recommended for most resected >T1N0 nmGaC to further improve survival, it may not be needed for T2-3 cancers without LN metastasis (10–12). Postsurgical radiotherapy may further enhance survival especially for disease with more advanced nodal stage or after a limited lymphadenectomy, while it has limited survival impact on node-negative disease (10–12).

For resected nmGaC, previous researches have revealed greatly controversial findings on the correlation between examined LN (ELN) count and long-term survival. While some studies (20, 21) revealed that more ELNs were associated with better survival even for N0 disease, a Korean study (22) and a study of Korean-American patients (23) did not suggest a significant association for all-stage cancers, and further studies (24, 25) showed that the association varied across different tumor factors. The discrepant biologic behaviors underlying the various tumor characteristics may impact the prognostic significance of LN examination.

ELN count is a vital metric for evaluation of quality in cancer care, whereas LN ratio (LNR) may not be as practical especially before knowing the exact positive LN (PLN) number. There has been no universally accepted minimal count of ELNs needed for accurate and reliable pathologic staging of nmGaC. While most guidelines (3–14) recommend a minimum of 15 or 16 LNs to be examined, the French Intergroup Clinical Practice Guidelines (15) propose retrieval of ≥25 LNs. Notably, these guideline recommendations are mostly based on small single-institution studies using univariable analysis to identify the cut-points without stratified analysis, which is not sufficiently robust and could be biased by other important confounders. The optimal count to satisfactorily stratify patient survival has yet to be established for nmGaC.

To tackle these unsettled issues, we herein analyzed two large cohorts from the West and the East including discrepant ethnicities, cancer characteristics, clinical routines, and patient outcomes (26), which may more vividly depict the real-world situations. We hoped to robustly clarify the correlations of ELN count with staging and survival in resected nmGaC, and to define the minimum and optimum threshold ELN numbers using a multivariable approach.

## Materials and Methods

### Patients

Individual-level data on patients with nmGaC were retrieved from the US Surveillance, Epidemiology, and End Results (SEER)-18 database and a Chinese multi-institutional gastric cancer database (MIGC). The SEER Program is an authoritative source of information on incidence and survival of cancer in the US, and collects data from population-based cancer registries covering ~35% of the US population. The MIGC, a central database managed and maintained by an independent biostatistician, recorded data of high quality on consecutive patients with nmGaC undergoing surgical resection at the department of gastrointestinal surgery of two Chinese high-volume tertiary institutions specialized in stomach surgery (First and Fourth Affiliated Hospital of Anhui Medical University), using a uniform standardized data-collecting form. This study was approved by the institutional review boards of the two institutions, and the need for informed consent was waived considering the anonymous data retrieval.

Only individuals with microscopically confirmed primary invasive TNM stage I-III adenocarcinoma of the stomach undergoing surgical resection in 2010 through 2016 were included (**Table S1**). Cancers of other histology types including squamous cell carcinoma, gastrointestinal stromal tumors/sarcoma, neuroendocrine tumor/carcinoid, lymphoma, and germ-cell tumor were ineligible (**Table S2**). Patients with non-gastric cancers involving the stomach, with benign or *in situ* tumors, or with ≥2 malignancies in their lifetimes were excluded. Those with diagnosis on the basis of autopsy or death certificate only or with missing follow-up period or survival status were also ineligible. Cancers with distant metastasis were not included since resection is not routinely recommended for them. Cases with 0 or unknown ELNs were ineligible, considering that lymphadenectomy is part of standard resection for nmGaC and that the ELN count is needed to be reported (10–12). Patients diagnosed before 2010 were not eligible, considering the incompatibility between the TNM staging editions in effect before and since 2010. To minimize the effect of perioperative events on survival, we excluded patients surviving <3 months. Cases with missing data were excluded from analysis (missing proportions of all study variables were <10%).

Data on patient (sex, age, diagnosis year, follow-up time, and survival status), tumor (morphology, topography, stage, ELN number, PLN number, differentiation, and size), and treatment variables (resection type and margin) were retrieved. Information on chemotherapy and radiotherapy was recorded with low sensitivity in SEER-18 (27) where neoadjuvant chemotherapy could not be differentiated from adjuvant chemotherapy. Data on immunotherapy were not available in SEER-18. Neoadjuvant therapy was not routinely performed in the China centers, and information on adjuvant chemotherapy, radiotherapy, and immunotherapy was not available in MIGC. Tumor morphology and topography were based on the International Classifications of Diseases for Oncology, Third Edition. Cancer stage was in accordance with the AJCC/UICC TNM staging system, Seventh Edition. The ELN count was the total number of regional LNs which were intraoperatively removed by surgeons and postoperatively examined by pathologists. LNR was computed by dividing PLN by ELN count.

### Statistics

Based on the hypothesis that sampling more LNs increases the probability of identifying more PLNs, stage migration was assessed by quantifying the correlation of ELN number with the proportion of positive *versus* negative nodal stage and of each declared N stage [N0 (reference), N1, N2, N3a, and N3b] using the binomial and multinomial logistic regression, respectively, with adjustment for other confounders possibly associated with ELN or PLN number before or during operation (diagnosis year, patient sex, age, cancer local invasion, differentiation, location, and type of resection). Overall survival time was calculated until death from any cause or last follow-up. The prognostic impact of ELN number was measured using multivariable Cox proportional hazards (PH) regression adjusting for other potential prognostic factors (diagnosis year, sex, age, tumor local invasion, PLN number, location, differentiation, resection type, and margin). Given the low sensitivity of the non-resection treatment variables (27) and the unavailability of time intervals between surgery and non-surgical management, in SEER-18 radiotherapy or chemotherapy was not included further in the multivariable models. Subgroup analyses were performed by stratifying the models by patient, tumor, and treatment factors listed in **Table 1**. In subgroup analyses, we added the corresponding category restriction in sex, age group, tumor invasion, local invasion, differentiation, size group, resection type, chemotherapy, or radiotherapy to the overall eligible patients. For example, when conducting subgroup analysis for male patients, we further restricted the overall eligible patients to males. The proportional hazards assumption was verified both graphically using the log-log plot and analytically using the scaled Schoenfeld residuals test before performing survival analyses (28).

**Table 1:** Demographic and clinicopathologic characteristics of patients with resected non-metastatic gastric adenocarcinoma and with ≥1 examined lymph node, 2010–2016^1^.

Parameter		SEER-18, the US	MIGC, China
**N**		7,228	1,468
**Sex**	Male	4,552 (63)	1,003 (68)
**Age** (years)	As continuous	65 ± 13, 66 (56–74)	56 ± 12, 57 (48–64)
	<50	895 (12)	408 (28)
	50–59	1,494 (21)	478 (33)
	60–69	2,048 (28)	209 (28)
	70–79	1,849 (26)	154 (10)
	≥80	942 (13)	19 (1)
**Tumor location**	Gastric cardia	1,998 (28)	336 (23)
	Gastric fundus/body	922 (13)	354 (24)
	Gastric antrum/pylorus	2,204 (30)	775 (53)
	Other^2^	2,104 (29)	3 (<1)
**Tumor local invasion**	Lamina propria/submucosa	1,686 (23)	215 (15)
	Muscularis propria/subserosa	3,903 (54)	296 (20)
	Serosa	1,232 (17)	772 (53)
	Adjacent structures	407 (6)	185 (13)
**Positive lymph node count**	As continuous	4 ± 7, 1 (0–5)	5 ± 7, 2 (0–6)
	0	3,256 (45)	601 (41)
	1–2	1,336 (18)	257 (18)
	3–6	1,187 (16)	270 (18)
	7–15	1,007 (14)	221 (15)
	≥16	442 (6)	119 (8)
**Examined lymph node count**	As continuous	20 ± 13, 17 (11–25)	30 ± 21, 24 (1–44)
**Lymph node ratio**	As continuous	0.20 ± 0.28, 0.06 (0.00–0.32)	0.19 ± 0.26, 0.06 (0.00–0.30)
**Tumor differentiation grade**	Well	386 (5)	147 (10)
	Intermediate	2,020 (28)	425 (29)
	Poor/undifferentiated	4,822 (67)	896 (61)
**Tumor size** (cm)	As continuous	4.6 ± 4.1; 4.0 (2.3–6.0)	4.1 ± 2.6; 4.0 (2.5–5.0)
	<2	1,249 (17)	175 (12)
	2–4	2,173 (30)	532 (36)
	4–6	1,881 (26)	460 (31)
	6–8	1,027 (14)	198 (13)
	≥8	898 (12)	103 (7)
**Resection type**	Partial/subtotal gastrectomy	5,067 (70)	1,026 (70)
	Total/near-total gastrectomy	1,585 (22)	416 (28)
	Gastrectomy (NOS)	576 (8)	26 (2)
**Resection margin**	Positive	NA	25 (2)
**Neoadjuvant chemotherapy**^3^	Yes	NA	0 (0)
**Neoadjuvant radiotherapy**^3^	Yes	939 (13)	0 (0)
**Adjuvant chemotherapy**^3^	Yes	4,327 (60)	NA
**Adjuvant radiotherapy**^3^	Yes	1,845 (26)	NA
**Accumulated follow-up**	Person-years	17,670	5,444
**Follow-up time**^4^	Months	41 (22–61)	52 (28–89)

^1^Enumeration data are shown as count (percentage [%]), and measurement data as mean ± standard deviation, median (interquartile range). Records are complete otherwise specified below.

^2^Lesser curvature, greater curvature, overlapping lesion of stomach, and stomach (NOS).

^3^In the US, neoadjuvant and adjuvant chemotherapy could not be differentiated from each other; the other category for the non-surgical variables was “No/unknown,” considering the low sensitivity.

^4^Shown as median (interquartile range), and computed using the reverse Kaplan-Meier method.

SEER, Surveillance, Epidemiology, and End Results Program; MIGC, the multi-institutional gastric cancer database; NOS, not otherwise specified; NA, not available.

The correlations of more ELNs with multivariable-adjusted odds ratios (ORs) for negative-to-positive and lower-to-higher nodal stage migration and hazard ratios (HRs) for survival, and with the mean PLN count and LNR were visualized using plots, which were smoothed with the use of the LOcally WEighted Scatterplot Smoothing (LOWESS) method with a default bandwidth of 2/3 (29). The most frequent ELN number in the US cohort (15) was applied as the reference. The structural breakpoints for the fitted variables in the total and patient subgroups were then determined using the Chow test (*F* test). Considering that survival outcomes are the most vital, the breakpoint for fitted HRs for survival in the overall derivation US cohort was deemed as the optimum threshold. To reliably declare a node-negative disease, the breakpoint for fitted ORs for negative-to-positive nodal stage migration in the US cohort was selected as the minimal threshold.

The threshold ELN numbers were validated by associating examining more LNs than the determined thresholds with staging and survival using multivariable-adjusted models in overall and stratified analyses. Multivariable-adjusted survival curves stratified by cut-off ELN number using the Cox model were illustrated. Time-dependent receiver operating characteristic curve (ROC) analysis was further done to evaluate the accuracy of the multivariable-adjusted Cox model incorporating the threshold-dichotomized ELN number in prediction of survival, and the area under the ROC curve (AUC) at each follow-up time and the integrated AUC (iAUC) across all the follow-up period were calculated following Heagerty and Zheng (30).

Analyses were done using the R 3.5.1 software (https://cran.r-project.org), with findings considered statistically significant at two-sided *P*<0.05.

## Results

### Patient Characteristics

Together 7,228 nmGaC patients from the US and 1,468 from China undergoing cancer-directed resection in 2010–2016 were eligible for analysis (**Table S1**). The median follow-up was 41 and 52 months and the accumulated follow-up was 17,670 and 5,444 person-years in the US and China cohorts, respectively (**Table 1**). In both cohorts, male patients comprised the majority (SEER-18, 63%; MIGC, 68%). The US patients were older than the China patients (mean age, 65 *vs* 56). Cancers most often invaded muscularis propria/subserosa in the US (54%) but serosa in China (53%). Most resected cancers were poorly differentiated/undifferentiated (SEER-18, 67%; MIGC, 61%), and had the size of 2–4 cm (SEER-18, 30%; MIGC, 36%). Partial/subtotal gastrectomy was most commonly performed (70% in both cohorts).

The mean ELN count was smaller in the US than in the China cohort (20 *vs* 30), while the mean PLN count was similar between the two cohorts (4 *vs* 5), with the majority of cases being node-negative (45% *vs* 41%).

### Examined Lymph Node Number and Staging

**Figure 1:**
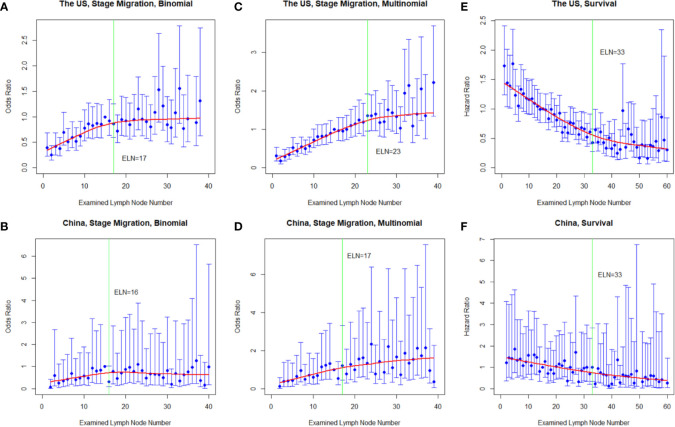
Multivariable-adjusted odds ratios (ORs) for negative-to-positive (binomial; **A**, **B**) and lower-to-higher nodal stage migration (multinomial, **(C**, **D)** and hazard ratios (HRs) for overall survival **(E, F)** with each additional examined lymph node (ELN) in resected gastric adenocarcinoma in the US and China cohorts. ORs were calculated using multivariable binomial/multinomial logistic regression adjusting for year of diagnosis, sex, age, tumor local invasion, differentiation, location, size, and resection type. HRs were computed using multivariable Cox proportional hazards regression adjusting for year of diagnosis, sex, age, tumor local invasion, differentiation, location, size, metastatic lymph node number, and resection type. The adjusted ORs and HRs and the corresponding 95% confidence intervals are shown in blue, and the smoothed curves fitted using the LOcally WEighted Scatterplot Smoothing (LOWESS) method with a default bandwidth of 2/3 are shown in red. The vertical green lines indicate the determined structural breakpoints using the Chow test.

With more ELNs, the identified PLN number increased while the LNR decreased in both cohorts (**Figure S1**). Consistently, using the multivariable-adjusted logistic models, more ELNs were significantly associated with proportional increases from node-negative to node-positive diseases (binomial; OR_SEER-18_ = 1.02, 95% confidence interval (CI) = 1.01–1.02, *P* < 0.001; OR_MIGC_ = 1.01, 95% CI = 1.00–1.02, *P* = 0.049) and from lower to higher nodal stages (multinomial; OR_SEER-18_ = 1.03, 95% CI = 1.03–1.04, *P* < 0.001; OR_MIGC_ = 1.02, 95% CI = 1.02–1.03, *P* < 0.001), and the multinomial associations remained significant in most stratifications by sex, age group, cancer location, local invasion, differentiation, size group, resection type, and administration of radiotherapy and chemotherapy (**Table 2**). With more ELNs, while the odds for identifying more advanced nodal stages increased, the increasing trend markedly weakened after certain ELNs (**Figures 1A–D**).

**Table 2:** Association of examined lymph node count (entered as continuous) with lower-to-higher nodal stage migration in resected non-metastatic gastric adenocarcinoma patients with ≥1 examined lymph node using *multivariable*-adjusted multinomial logistic regression, overall and in *subgroups* by patient, tumor, and treatment factors^1^.

Stratification	The US			China			
	Adj. OR^1^	95% CI	*P_OR_*	Adj. OR^1^	95% CI	*P_OR_*	
**Overall**	1.03	1.03–1.04	**<0.001**	1.02	1.02–1.03	**<0.001**	
**Sex**							
Male	1.03	1.03–1.04	**<0.001**	1.02	1.01–1.03	**<0.001**	
Female	1.04	1.03–1.04	**<0.001**	1.03	1.02–1.04	**<0.001**	
**Age group**							
<50 years	1.03	1.02–1.04	**<0.001**	1.02	1.01–1.03	**0.002**	
50–59 years	1.03	1.02–1.04	**<0.001**	1.02	1.01–1.03	**0.002**	
60–69 years	1.04	1.03–1.04	**<0.001**	1.03	1.02–1.04	**<0.001**	
70–79 years	1.03	1.02–1.04	**<0.001**	1.04	1.01–1.07	**0.012**	
≥80 years	1.05	1.04–1.06	**<0.001**	NE	NE	NE	
**Tumor location**							
Gastric cardia	1.03	1.02–1.04	**<0.001**	1.03	1.02–1.05	**<0.001**	
Gastric fundus/body	1.03	1.02–1.03	**<0.001**	1.02	1.01–1.04	**<0.001**	
Gastric antrum/pylorus	1.05	1.04–1.05	**<0.001**	1.02	1.01–1.03	**<0.001**	
Other^2^	1.03	1.03–1.04	**<0.001**	NA	NA	NA	
**Tumor local invasion**							
Lamina propria/submucosa	1.02	1.01–1.03	**<0.001**	1.02	0.99–1.04	0.186	
Muscularis propria/subserosa	1.03	1.03–1.03	**<0.001**	1.01	1.00–1.03	0.152	
Serosa	1.05	1.04–1.06	**<0.001**	1.03	1.02–1.04	**<0.001**	
Adjacent structures	1.06	1.04–1.07	**<0.001**	1.04	1.02–1.06	**<0.001**	
**Differentiation**							
Well	1.03	1.01–1.06	**0.017**	1.04	1.01–1.07	**0.010**	
Intermediate	1.03	1.02–1.04	**<0.001**	1.01	1.00–1.02	0.242	
Poor/undifferentiated	1.04	1.03–1.04	**<0.001**	1.03	1.02–1.04	**<0.001**	
**Tumor size group**							
<2 cm	1.02	1.01–1.04	**<0.001**	1.04	1.00–1.07	**0.037**	
2–4 cm	1.03	1.02–1.04	**<0.001**	1.01	1.00–1.02	**0.040**	
4–6 cm	1.04	1.03–1.04	**<0.001**	1.02	1.01–1.04	**<0.001**	
6–8 cm	1.04	1.03–1.05	**<0.001**	1.04	1.02–1.06	**<0.001**	
≥8 cm	1.04	1.03–1.05	**<0.001**	1.03	1.01–1.06	**0.013**	
**Resection type**							
Partial/subtotal gastrectomy	1.04	1.03–1.04	**<0.001**	1.02	1.01–1.03	**<0.001**	
Total/near-total gastrectomy	1.03	1.02–1.04	**<0.001**	1.03	1.02–1.04	**<0.001**	
Gastrectomy (NOS)	1.03	1.02–1.05	**<0.001**	NE	NE	NE	
**Chemotherapy, yes**	1.03	1.03–1.04	**<0.001**	NA	NA	NA	
**Radiotherapy, yes**	1.05	1.04–1.05	**<0.001**	NA	NA	NA	

^1^Odds ratios for association of examined lymph node count with serial advancement in nodal stage overall and in stratifications were computed using multivariable multinomial logistic regression models adjusting for year of diagnosis, sex, age, tumor location, local invasion, differentiation, size, and resection type. Subgroup analyses were performed by stratifying the models by patient, tumor, and treatment factors listed in **Table 1**. In subgroup analyses, we added the corresponding category restriction in sex, age group, tumor invasion, local invasion, differentiation, size group, resection type, chemotherapy, or radiotherapy to the overall eligible patients. For example, when conducting subgroup analysis for male patients, we further restricted the overall eligible patients to males. Statistically significant P values are shown in bold.

^2^Lesser curvature, greater curvature, overlapping lesion of stomach, and stomach (NOS).

Adj., adjusted; OR, odds ratio; CI, confidence interval; NOS, not otherwise specified; NE, not estimable due to small case number; NA, not available.

### Examined Lymph Node Number and Survival

After multivariable adjustment for the other prognostic factors, more ELNs were associated with better survival in both countries (HR_SEER-18_ = 0.97, 95% CI = 0.97–0.97, *P* < 0.001; HR_MIGC_ = 0.98, 95% CI = 0.97–0.99, *P* < 0.001). The associations remained significant and the reductions in mortality risk were very similar in most subgroups stratified by sex, age group, cancer location, local invasion, PLN group, differentiation, size group, resection type, and administration of chemotherapy and radiotherapy (**Table 3**). Notably, similar survival associations were observed even in declared node-negative disease (HR_SEER-18_ = 0.97, 95% CI = 0.97–0.98, *P* < 0.001; HR_MIGC_ = 0.98, 95% CI = 0.96–1.00, *P* = 0.091). The HRs for mortality decreased with each additional ELN, but the decreasing trend dramatically weakened after certain ELNs (**Figures 1E, F**).

**Table 3:** Association of examined lymph node count (entered as continuous) with overall survival in resected non-metastatic gastric adenocarcinoma patients with ≥1 examined lymph node using *multivariable*-adjusted Cox proportional hazards regression, overall and in *subgroups* by patient, tumor, and treatment factors^1^.

Stratification	The US			China		
	Adj. HR^1^	95% CI	*P_HR_*	Adj. HR^1^	95% CI	*P_HR_*
**Overall**	0.97	0.97–0.97	**<0.001**	0.98	0.97–0.99	**<0.001**
**Sex**						
Male	0.97	0.97–0.98	**<0.001**	0.98	0.97–0.99	**<0.001**
Female	0.96	0.96–0.97	**<0.001**	0.98	0.97–1.00	**0.013**
**Age group**						
<50 years	0.97	0.96–0.98	**<0.001**	0.97	0.95–0.99	**0.006**
50–59 years	0.97	0.96–0.98	**<0.001**	0.97	0.96–0.99	**0.001**
60–69 years	0.97	0.96–0.98	**<0.001**	0.98	0.97–1.00	**0.014**
70–79 years	0.97	0.97–0.98	**<0.001**	0.99	0.96–1.02	0.494
≥80 years	0.97	0.96–0.98	**<0.001**	NE	NE	NE
**Tumor location**						
Gastric cardia	0.97	0.96–0.98	**<0.001**	0.98	0.96–1.00	**0.025**
Gastric fundus/body	0.98	0.97–0.99	**<0.001**	0.98	0.96–0.99	**0.003**
Gastric antrum/pylorus	0.97	0.96–0.97	**<0.001**	0.98	0.97–1.00	**0.006**
Other^2^	0.97	0.96–0.97	**<0.001**	NA	NA	NA
**Tumor local invasion**						
Lamina propria/submucosa	0.98	0.96–0.99	**<0.001**	0.95	0.89–1.03	0.198
Muscularis propria/subserosa	0.97	0.97–0.98	**<0.001**	0.98	0.95–1.01	0.127
Serosa	0.97	0.96–0.97	**<0.001**	0.98	0.97–0.99	**<0.001**
Adjacent structures	0.96	0.95–0.97	**<0.001**	0.98	0.96–1.00	**0.018**
**Positive lymph node count**						
0	0.97	0.97–0.98	**<0.001**	0.98	0.96–1.00	0.091
1–2	0.97	0.96–0.97	**<0.001**	0.99	0.97–1.01	0.361
3–6	0.97	0.96–0.98	**<0.001**	0.97	0.95–0.99	**0.001**
7–15	0.98	0.97–0.99	**<0.001**	0.98	0.97–1.00	**0.039**
≥16	0.96	0.95–0.98	**<0.001**	0.97	0.95–0.99	**0.013**
**Differentiation**						
Well	0.97	0.94–0.99	**0.002**	0.95	0.92–0.99	**0.016**
Intermediate	0.97	0.97–0.98	**<0.001**	0.99	0.97–1.00	0.083
Poor/undifferentiated	0.97	0.96–0.97	**<0.001**	0.98	0.97–0.99	**<0.001**
**Tumor size group**						
<2 cm	0.98	0.96–0.99	**<0.001**	0.99	0.94–1.03	0.529
2–4 cm	0.97	0.97–0.98	**<0.001**	0.97	0.96–0.99	**0.001**
4–6 cm	0.97	0.96–0.97	**<0.001**	0.98	0.97–0.99	**0.004**
6–8 cm	0.96	0.95–0.97	**<0.001**	0.98	0.96–1.00	**0.019**
≥8 cm	0.97	0.96–0.98	**<0.001**	0.99	0.96–1.01	0.268
**Resection type**						
Partial/subtotal gastrectomy	0.97	0.96–0.97	**<0.001**	0.98	0.97–0.99	**<0.001**
Total/near-total gastrectomy	0.97	0.97–0.98	**<0.001**	0.98	0.97–1.00	**0.015**
Gastrectomy (NOS)	0.98	0.96–0.99	**<0.001**	NE	NE	NE
**Chemotherapy, yes**	0.97	0.96–0.97	**<0.001**	NA	NA	NA
**Radiotherapy, yes**	0.97	0.97–0.98	**<0.001**	NA	NA	NA

^1^Hazard ratios for associations of examined lymph node count with overall survival were calculated by Cox proportional hazards regression adjusting for year of diagnosis, sex, age, tumor location, local invasion, differentiation, size, metastatic lymph node number, and resection type. Subgroup analyses were performed by stratifying the models by patient, tumor, and treatment factors listed in **Table 1**. In subgroup analyses, we added the corresponding category restriction in sex, age group, tumor invasion, local invasion, differentiation, size group, resection type, chemotherapy, or radiotherapy to the overall eligible patients. For example, when conducting subgroup analysis for male patients, we further restricted the overall eligible patients to males. Statistically significant P values are shown in bold.

^2^Lesser curvature, greater curvature, overlapping lesion of stomach, and stomach (NOS).

Adj., adjusted; HR, hazard ratio; CI, confidence interval; NOS, not otherwise specified; NE, not estimable due to small case number; NA, not available.

### Examined Lymph Node Number Thresholds

The fitting curves and determined structural breakpoints for the ORs for binomial and multinomial stage migration, the HRs for survival, the mean PLN number, and the mean LNR value with each additional ELN are shown in **Figures 1** and **S1**. The breakpoints for the above variables in the total cohorts and for the HRs in various subgroups by patient, tumor, and treatment factors are shown in **Table 4**. Because survival is the most important endpoint and for generalizability and representativeness, the structural breakpoint for survival in the US (33 ELNs) was selected as the optimal threshold. The breakpoints for HRs in subgroups by sex, age group, tumor location, local invasion, PLN group, differentiation, size group, resection type, and administration of chemotherapy and radiotherapy were mostly in accordance with each other with a few exceptions (**Table 4**). Cancers invading lamina propria/submucosa (22) and those without observed LN involvement (15) had markedly smaller breakpoints.

**Table 4:** Structural breakpoints of examined lymph node count based on different parameters and based on hazard ratio for overall survival in different subgroups^1^.

Parameter/stratification	Comment/category	Structural breakpoint	*F*	*P*^2^
*Based on different parameters*				
**Hazard ratio for overall survival**	The US	33	1,427.3	**<0.001**
**Hazard ratio for overall survival**	China	33	1,466.5	**<0.001**
**Odds ratio for lower-to-higher nodal stage migration, multinomial**	The US	23	854.1	**<0.001**
**Odds ratio for lower-to-higher nodal stage migration, multinomial**	China	17	656.3	**<0.001**
**Odds ratio for negative-to-positive nodal stage migration, binomial**	The US	17	1,112.4	**<0.001**
**Odds ratio for negative-to-positive nodal stage migration, binomial**	China	16	2,976.5	**<0.001**
**Positive lymph node number**	The US	23	1,892.1	**<0.001**
**Positive lymph node number**	China	18	481.0	**<0.001**
**Lymph node ratio**	The US	21	1,538.9	**<0.001**
**Lymph node ratio**	China	16	284.8	**<0.001**
***Based on hazard ratio for overall survival in different stratifications in the US***				
**Sex**	Male	30	1,659.8	**<0.001**
	Female	32	977.1	**<0.001**
**Age group**	<70 years	31	816.5	**<0.001**
	≥70 years	32	2,317.7	**<0.001**
**Tumor location**	Gastric cardia	31	1,936.2	**<0.001**
	Gastric fundus/body/antrum/pylorus	30	536.7	**<0.001**
**Tumor local invasion**	Lamina propria/submucosa	22	416.5	**<0.001**
	Muscularis propria/subserosa	27	1,539.6	**<0.001**
	Serosa/adjacent structures	30	963.3	**<0.001**
**Positive lymph node count**	0	15	1,782.6	**<0.001**
	≥1	32	1,198.3	**<0.001**
**Differentiation**	Well/intermediate	32	307.5	**<0.001**
	Poor/undifferentiated	31	1,311.1	**<0.001**
**Tumor size group**	<4 cm	31	690.6	**<0.001**
	≥4 cm	30	1,683.5	**<0.001**
**Resection type**	Partial/subtotal gastrectomy	31	893.9	**<0.001**
	Total/near-total gastrectomy	30	1,510.8	**<0.001**
	Gastrectomy (NOS)	29	885.9	**<0.001**
**Chemotherapy**	Yes	31	1,444.8	**<0.001**
**Radiotherapy**	Yes	29	1,491.5	**<0.001**

^1^Results are derived from the US cohort if not otherwise specified in the “Comment/category” column. Structure breakpoints were determined using the Chow test for the LOWESS smoother-fitted associations of examined lymph node count with the indicated parameters overall and in subgroups. Odds ratios for association of examined lymph node count with positive versus negative nodal status and serial advancement in nodal stage overall and in subgroups were computed using multivariable binomial and multinomial logistic regression models, respectively, adjusting for year of diagnosis, sex, age, tumor location, local invasion, differentiation, size, and resection type. Hazard ratios for associations of examined lymph node count with overall survival were calculated using Cox proportional hazards regression adjusting for year of diagnosis, sex, age, tumor location, local invasion, differentiation, size, metastatic lymph node number, and resection type. Subgroup analyses were performed by stratifying the models by patient, tumor, and treatment factors listed in **Table 1**. In subgroup analyses, we added the corresponding category restriction in sex, age group, tumor invasion, local invasion, differentiation, size group, resection type, chemotherapy, or radiotherapy to the overall eligible patients. For example, when conducting subgroup analysis for male patients, we further restricted the overall eligible patients to males. Statistically significant P values are shown in bold.

^2^The P values are for the Chow Test (F Test) at the given structural breakpoints.

SEER, Surveillance, Epidemiology, and End Results Program; NOS, not otherwise specified; LOWESS, LOcally WEighted Scatterplot Smoothing.

**Figure 2:**
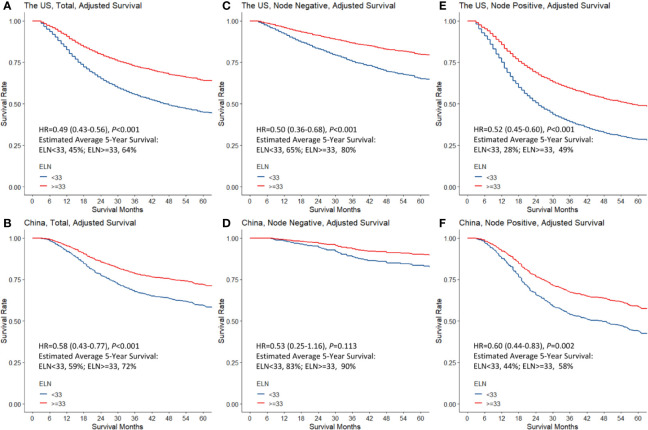
Stratification of adjusted overall survival by the determined optimal breakpoint of examined lymph node (ELN) number (33) in total patients with resected non-metastatic gastric adenocarcinoma **(A, B)**, and in patients with node-negative **(C, D)** and node-positive diseases **(E, F)** in the US and China cohorts. Multivariable Cox proportional hazards regression was used adjusting for year of diagnosis, sex, age, tumor local invasion, differentiation, location, size, and resection type. Positive lymph node count was also adjusted for in overall and node-positive cases. HR, hazard ratio.

**Figure 3:**
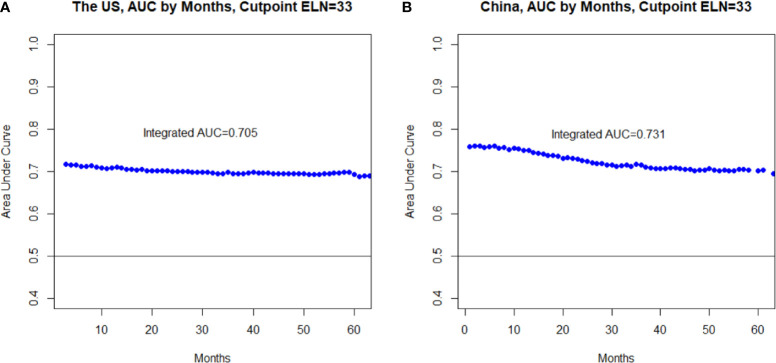
Area under the time-dependent receiver operating characteristic curve (AUC) at each follow-up time **(A)** and the integrated AUC (iAUC) across all the follow-up period **(B)** assessing the accuracy of the multivariable-adjusted Cox proportional hazards regression model incorporating the optimal threshold (33)-dichotomized ELN number in survival prediction.

The determined optimal threshold was validated both internally in the derivative US cohort and externally in the independent China cohort: After multivariable adjustment, in both cohorts examining ≥33 LNs was significantly associated with both lower risks of all-cause mortality (HR_SEER-18_ = 0.49, 95% CI = 0.43–0.56, *P* < 0.001; HR_MIGC_ = 0.58, 95% CI = 0.43–0.77, *P* < 0.001; **Figure 2**) and greater odds for detecting higher nodal stages (multinomial; OR_SEER-18_ = 2.21, 95% CI = 1.95–2.52, *P* < 0.001; OR_MIGC_ = 1.69, 95% CI = 1.29–2.21, *P* < 0.001) compared to screening <33 nodes. The association patterns remained similar in most subgroups by patient, cancer, and management factors (**Tables S3**, **4**). Although the survival correlation was still significant in declared node-negative nmGaCs in the US (HR = 0.50, 95% CI = 0.36–0.68, *P* < 0.001), it was not significant in node-positive tumors in China (HR = 0.53, 95% CI = 0.25–1.16, *P* = 0.113; **Figure 2**). The iAUC for the multivariable Cox model incorporating ELN count dichotomized by 33 was 0.705 in the US and 0.731 in the China cohort, and the AUC per time-point remained relatively stable across follow-up periods (**Figure 3**). Changes of HR, OR (both binomial and multinomial), and mean PLN number with more ELNs all markedly became less steep with ≥33 ELNs in both cohorts (**Figures 1** and **S1**).

To enable a reliable claim of node-negative disease, 17 ELNs were further chosen as the minimum threshold on the basis of the ORs for negative-to-positive nodal stage migration in the US (**Table 4**), which was validated in a way similar to that for the optimum threshold in both overall (survival: HR_SEER-18_ = 0.62, 95% CI = 0.57–0.67, *P* < 0.001; HR_MIGC_ = 0.69, 95% CI = 0.54–0.87, *P* = 0.002; multinomial stage migration: OR_SEER-18_ = 2.03, 95% CI = 1.85–2.23, *P* < 0.001; OR_MIGC_ = 1.71, 95% CI = 1.31–2.24, *P* < 0.001) and stratified analyses.

## Discussion

In our large observational study, multivariable-adjusted stage migration analyses of resected nmGaC patients from the US and China revealed that more ELNs were associated with more PLNs detected and larger proportions of greater nodal stages identified in the whole cohorts and in various subgroups stratified by patient, tumor, and treatment variables, suggesting more accurate staging. Both cohorts also consistently presented better long-term survival associated with more ELNs both overall and in all nodal stages in analyses adjusting for multiple variables including PLN number. The association findings were not significant in a few categories in the China cohort, possibly due to the paucity of cases. LNR was not always a stable parameter with varying ELNs. We further determined the minimal and optimal threshold ELN numbers to be 17 and 33, respectively, and validated them in both cohorts with good potential to differentiate probabilities of both stage migration and survival.

The different mean ELN numbers between the US (20) and China cohorts (30) could reflect the practice discrepancies between the West and the East. In the US in which gastric adenocarcinoma incidence is much lower, most of the patients are managed at non-referral institutions with limited lymphadenectomy (*e.g.*, D0/1), while Western surgeons have increasingly accepted the importance of doing more than a D1 node dissection (31). In high-incidence Eastern Asian countries including China, more extensive lymphadenectomies (*e.g.*, D2) are standard.

The observed survival associations do NOT suggest causality, and could possibly be explained by several reasons, prominently stage migration (32). Among patients with fewer ELNs, some with declared lower nodal stage could be mistakenly staged due to insufficient LN sampling and may have actually more advanced diseases, which causes relatively poorer observed survival of the patient subgroup. Sampling more nodes could increase the possibility of correctly identifying node-positive diseases and cancers with more advanced nodal stage which strongly necessitates the application of adjuvant chemotherapy, thus guarantying the adequate administration of postsurgical treatment for those who indeed need them. The possibility that patients with node-positive disease inappropriately do not undergo further postsurgical management due to cancers being misdiagnosed as node-negative as a result of fewer examined nodes is reduced. Removing more LNs may contribute to more thorough clearance of possible malignant remnants (source of recurrence), which enhances long-term survival. The ELN number could also somehow indicate the expertise of the treating center within a population-based setting, and more ELNs are more often achieved in larger specialized institutions where survival is usually better.

Current recommendations on ELN cutoff number in nmGaC vary across guidelines (3–15). While some studies (19–21, 33–36) have attempted to set up a benchmark and suggested cutoffs varying greatly from 10 to 40 ELNs, the methods used were mostly univariable with lack of robustness. Using a multivariable approach, we determined and validated the minimal and optimal cutoff ELN numbers for overall resected nmGaC, which were agreed by both cohorts albeit with discrepant practice patterns and which can be an effective quality metric and reference for defining adequate LN dissection. In both cohorts significant and independent associations of ≥17 or ≥33 ELNs with reduced risk of mortality especially in node-positive cancer and with more correct staging were observed. Meeting the minimal threshold ELN number determined based on negative-to-positive nodal stage migration could enable a more confident postsurgical declaration of N0 disease, and the optimal threshold nicely stratified postsurgical patient survival, which can be a reference for postsurgical therapy.

Notably, the determined minimal threshold precisely agrees with most guideline recommendations (3–14) which emphasize stage migration only and the average number of LNs harvested during limited lymphadenectomy (D1) (37), while the optimal threshold coincides well with the average node number for a more extensive approach (D2) (37). The adequate extent of lymphadenectomy in nmGaC remains debatable, and the increasing rates of postoperative morbidity and mortality are an outstanding concern for more extended lymphadenectomy, which possibly compromise long-term survival (31, 37–39). However, if the postsurgical events are avoided, D2 lymphadenectomy could be beneficial (38, 40, 41). Consistently, in our study excluding patients surviving <3 months, the hazards for mortality sequentially decreased until 33 ELNs. Encouragingly, surgeon experience and surgery quality have been improving with reducing postoperative events (42, 43). An Asian randomized trial (41) without operative death further reported the survival benefits of D3 lymphadenectomy compared to D1 dissection. While we observed a further slight decrease in mortality after 33 ELNs, the decreasing trend was dramatically weaker, and it is likely that surgical safety and postsurgical quality of life markedly decreases with further LN dissection especially when by unexperienced hands. The increase in postsurgical events and potential survival benefit associated with more ELNs should be well-balanced. Too many ELNs may even hamper prognosis, and there can be a maximum threshold for ELN count after which the death hazard may dramatically rise. However, this could not be addressed in our study due to the small case number with larger ELN counts. Our findings do NOT encourage more extended lymphadenectomy in nmGaC before further randomized data are obtained.

Although mostly slightly, the thresholds could vary according to different patient and tumor factors. Less extensive gastrectomy may already provide sufficient favorable benefits to and thus be plausible for cancers of earlier stage, and consistently we found that the thresholds for cancers with invasion limited to lamina propria/submucosa or without declared positive nodes were markedly lower, which agreed more with a D1 procedure. Notably, pre- and intraoperative staging can be unreliable and vary in accuracy across countries. It is still undecided whether the benefit of stage migration could be directly translated into enhanced clinical outcomes.

The determined threshold may not be uniformly optimal for each individual patient, and it may be challenging to achieve the threshold for some patient subpopulations despite optimal surgery and pathologic evaluation and in low-volume centers. Western patients may be more challenging to manage due to being more often obese and aged.

This study was first limited by the observational nature. Findings from observational investigations can only suggest associations, but cannot infer causality. It CANNOT be concluded that dissection or examination of more nodes benefits survival. Some other prognostic factors (*e.g.*, comorbidities) are not accounted for. The associations of ELN number with chemotherapy, radiotherapy, and immunotherapy could not be assessed in this study due to being either registered with low sensitivity or not available, and should be addressed in further studies. Based on the relatively high specificity of recording data on chemotherapy and radiotherapy in SEER-18 (27), we performed subgroup analyses for patients receiving chemotherapy and those receiving radiotherapy, and the results were similar to those from analyses of all eligible patients. Information on LN number by station was not available. As prevalence of LN involvement in different stations may vary, a better understanding of the role of each station could contribute to more precise management. However, a study of more than 1,000 patients did not suggest a significant prognostic relevance for the location of PLNs, rather, the number best defines prognosis (16).

The ultimate ELN number depends on the cooperation between surgeons dissecting specimens and pathologists identifying nodes. The enumeration methods may vary across centers in the population-based US cohort. Nevertheless, results from the tertiary Chinese institutions following identical surgical and pathological protocols were similar to the US findings. The incomplete separation and possible fragmentation of nodes could bias the reported ELN number and limit the application of the thresholds. The ELN count may also be influenced by other factors including anatomic variation and patient immune status (44, 45). Since it is hardly possible to fully verify a node-negative cancer before surgery, patients irrespective of nodal status were initially included, with further stratification analyses by nodal stage performed. Our report may serve as a pointer for further investigation, hopefully including patients with the same level of lymphadenectomy, with information on LN number by station, and with clear and analyzable data on neoadjuvant chemotherapy and neoadjuvant radiotherapy, especially in terms of survival.

The majority of the *P* values were <0.001 in statistical analyses especially in the US SEER-18 cohort. The relatively large sample sizes of the overall and stratified US cohorts made the confidence intervals narrow, with small *P* values. Normally if the results are significant in nature, larger sample sizes could result in narrower confidence intervals and smaller *P* values (46). In the subgroup analysis of well differentiated cancers in the US cohort with relatively smaller sample size (n = 386) for example and in some China subgroups, the *P* values were larger (**Tables 2**–**4**). The clinical significance would not be merely reflected by the statistical significance, and could be somehow assessed based on the OR and HR estimates.

To the best of our knowledge, this investigation is the largest to determine the clinicopathologic impact and both the minimal and optimal thresholds of ELNs in nmGaC, analyzing international multicenter real-world patient-level data and using robust statistics. The results based on both Western and Eastern cohorts are well representative and generalizable.

## Conclusion

More ELNs are correlated with more accurate nodal staging, which could partly account for the correlation with better survival in resected nmGaC in this observational investigation, where NO confirmative conclusions on causation or benefits could be made. Our findings robustly conclude 17 ELNs as the minimum and suggest 33 ELNs as the possible optimum thresholds for nmGaC patients expected to survive ≥3 months postoperatively, for the overall quality evaluation of clinical LN examination and for the stratification of postsurgical patient survival especially in observed node-positive disease. Our report may provide crucial references for determining population-based quality metrics in nmGaC management.

## Data Availability Statement

The Surveillance, Epidemiology, and End Results (SEER) Program data are available upon reasonable request and with permission of the registry. Restrictions apply to the availability of the Chinese data that support the findings of this study, which were used under license for the current study, and so are not publicly available.

## Ethics Statement

The studies involving human participants were reviewed and approved by the First and Fourth Affiliated Hospital of Anhui Medical University. Written informed consent was obtained from all the patients before registration in the Chinese database.

## Author Contributions

LH and AX conceptualized or designed the study. LH, XZ, and ZW acquired, analyzed, or interpreted the data. LH drafted the manuscript. XZ, ZW, and AX critically revised the manuscript for important intellectual content. LH conducted the statistical analysis. AX provided administrative, technical, or material support. All authors contributed to the article and approved the submitted version.

## Funding

This study was supported by the National Natural Science Foundation of China (Grant No.: 81572350). The funders had no involvement in study design; in the collection, analysis, or interpretation of data; in the writing of the report; or in the decision to submit the paper for publication.

## Conflict of Interest

The authors declare that the research was conducted in the absence of any commercial or financial relationships that could be construed as a potential conflict of interest.
