# Supplementary material for: Importance of Examined Lymph Node Number in Accurate Staging and Enhanced Survival in Resected Gastric Adenocarcinoma—The More, the Better? A Cohort Study of 8,696 Cases From the US and China, 2010–2016
Source: Front Oncol. 2021 Jan 6;10:539030. doi: 10.3389/fonc.2020.539030 (PMC7874152; doi:10.3389/fonc.2020.539030)
Supplement: Supplementary file 1 [file Table_1.docx]

**Supplementary material**

**Table S1.** General information on the US and China gastric adenocarcinoma cohorts

**Table S2.** Inclusion and exclusion codes according to International Classification of Diseases for Oncology, Third Edition

**Table S3.** Association of ≥ versus < 33 examined lymph nodes with overall survival in resected non-metastatic gastric adenocarcinoma patients with ≥ 1 examined lymph node using *multivariable*-adjusted Cox proportional hazards regression, overall and in *subgroups* by patient, tumor, and treatment factors

**Table S4.** Association of ≥ versus < 33 examined lymph nodes with lower-to-higher nodal stage migration in resected non-metastatic gastric adenocarcinoma patients with ≥ 1 examined lymph node using *multivariable*-adjusted multinomial logistic regression, overall and in *subgroups* by patient, tumor, and treatment factors

**Figure S1.** Associations of examined lymph node number (ELN) with positive lymph node number and lymph node ratio in the US and China cohorts.

**Table S1.** General information on the US and China gastric adenocarcinoma cohorts

| Source | Country | Diagnosis period | Censoring date | Resected microscopically-confirmed primary cases with eligible histology and without missing variables, 2010 or later | Excluded cases^1^ | | | Eligible cases with ≥ 1 ELN |
| --- | --- | --- | --- | --- | --- | --- | --- | --- |
|  |  |  |  |  | Survival unknown/  <3 months | Stage 0/IV/unknown | 0 ELNs |  |
| SEER-18 | The US | Jan. 2010-Dec. 2016 | Dec. 31, 2016 | 9205 | 873 (9) | 704 (8) | 400 (4) | 7228 |
| MIGC | China | Jan. 2010-Dec. 2016 | Dec. 31, 2016 | 2101 | 213 (10) | 368 (18) | 52 (2) | 1468 |

^1^Shown as n (percentage [%]).

SEER, Surveillance, Epidemiology, and End Results Program; MIGC, the multi-institutional gastric cancer database; ELN, examine lymph node.

**Table S2.** Inclusion and exclusion codes according to International Classification of Diseases for Oncology, Third Edition

| **Category** |  | **Code** |
| --- | --- | --- |
| **Topology** | Inclusion | C16, C16.0, C16.1, C16.2, C16.3, C16.4, C16.5, C16.6, C16.8, C16.9 |
|  | Exclusion | - |
| **Morphology** | Inclusion | 8000-8089, 8140-8389, 8440-8579 |
|  | Exclusion | 8013, 8052, 8053, 8060, 8070-8078, 8081, 8083, 8084, 8152, 8153, 8156, 8160, 8170, 8240-8243, 8246, 8249, 8252 |
| **Behavior** | Inclusion | 3 (invasive) |
|  | Exclusion | 0 (benign), 2 (*in situ*) |

**Table S3.** Association of ≥ versus < 33 examined lymph nodes with overall survival in resected non-metastatic gastric adenocarcinoma patients with ≥ 1 examined lymph node using *multivariable*-adjusted Cox proportional hazards regression, overall and in *subgroups* by patient, tumor, and treatment factors^1^

| **Stratification** | **The US** | | |  | **China** | | |
| --- | --- | --- | --- | --- | --- | --- | --- |
|  | **Adj. HR**^1^ | **95% CI** | ***P_HR_*** |  | **Adj. HR**^1^ | **95% CI** | ***P_HR_*** |
| **Overall** | 0.49 | 0.43-0.56 | **< 0.001** |  | 0.58 | 0.43-0.77 | **< 0.001** |
| **Sex** |  |  |  |  |  |  |  |
| Male | 0.54 | 0.46-0.64 | **< 0.001** |  | 0.63 | 0.45-0.89 | **0.009** |
| Female | 0.42 | 0.34-0.52 | **< 0.001** |  | 0.41 | 0.23-0.74 | **0.003** |
| **Age group** |  |  |  |  |  |  |  |
| < 50 years | 0.48 | 0.33-0.70 | **< 0.001** |  | 0.47 | 0.25-0.87 | **0.017** |
| 50-59 years | 0.53 | 0.40-0.71 | **< 0.001** |  | 0.50 | 0.29-0.88 | **0.016** |
| 60-69 years | 0.43 | 0.33-0.56 | **< 0.001** |  | 0.59 | 0.34-1.04 | 0.070 |
| 70-79 years | 0.51 | 0.39-0.65 | **< 0.001** |  | 0.64 | 0.27-1.53 | 0.315 |
| ≥ 80 years | 0.51 | 0.36-0.71 | **< 0.001** |  | NE | NE | NE |
| **Tumor location** |  |  |  |  |  |  |  |
| Gastric cardia | 0.51 | 0.39-0.67 | **< 0.001** |  | 0.68 | 0.37-1.26 | 0.223 |
| Gastric fundus/body | 0.65 | 0.47-0.89 | **0.008** |  | 0.54 | 0.31-0.95 | **0.032** |
| Gastric antrum/pylorus | 0.39 | 0.30-0.51 | **< 0.001** |  | 0.47 | 0.31-0.71 | **< 0.001** |
| Other^2^ | 0.49 | 0.40-0.60 | **< 0.001** |  | NA | NA | NA |
| **Tumor local invasion** |  |  |  |  |  |  |  |
| Lamina propria/submucosa | 0.45 | 0.26-0.78 | **0.004** |  | 0.94 | 0.15-5.86 | 0.947 |
| Muscularis propria/subserosa | 0.53 | 0.45-0.63 | **< 0.001** |  | 0.35 | 0.11-1.08 | 0.068 |
| Serosa | 0.47 | 0.37-0.59 | **< 0.001** |  | 0.67 | 0.46-0.97 | **0.034** |
| Adjacent structures | 0.42 | 0.27-0.63 | **< 0.001** |  | 0.48 | 0.27-0.85 | **0.012** |
| **Positive lymph node count** |  |  |  |  |  |  |  |
| 0 | 0.50 | 0.36-0.68 | **< 0.001** |  | 0.53 | 0.25-1.16 | 0.113 |
| 1-2 | 0.42 | 0.29-0.60 | **< 0.001** |  | 0.46 | 0.19-1.14 | 0.093 |
| 3-6 | 0.53 | 0.39-0.72 | **< 0.001** |  | 0.54 | 0.28-1.04 | 0.064 |
| 7-15 | 0.60 | 0.48-0.76 | **< 0.001** |  | 0.56 | 0.33-0.95 | **0.032** |
| ≥ 16 | 0.56 | 0.42-0.74 | **< 0.001** |  | 0.67 | 0.34-1.33 | 0.255 |
| **Differentiation** |  |  |  |  |  |  |  |
| Well | 0.30 | 0.10-0.93 | **0.037** |  | 0.57 | 0.16-2.08 | 0.398 |
| Intermediate | 0.40 | 0.30-0.55 | **< 0.001** |  | 0.77 | 0.44-1.36 | 0.371 |
| Poor/undifferentiated | 0.51 | 0.44-0.59 | **< 0.001** |  | 0.50 | 0.35-0.71 | **< 0.001** |
| **Tumor size group** |  |  |  |  |  |  |  |
| < 2 cm | 0.60 | 0.36-0.98 | **0.042** |  | 1.25 | 0.26-6.05 | 0.783 |
| 2-4 cm | 0.54 | 0.40-0.71 | **< 0.001** |  | 0.41 | 0.23-0.74 | **0.003** |
| 4-6 cm | 0.46 | 0.36-0.59 | **< 0.001** |  | 0.47 | 0.28-0.80 | **0.005** |
| 6-8 cm | 0.43 | 0.32-0.58 | **< 0.001** |  | 0.64 | 0.33-1.23 | 0.180 |
| ≥ 8 cm | 0.55 | 0.43-0.71 | **< 0.001** |  | 0.74 | 0.32-1.72 | 0.476 |
| **Resection type** |  |  |  |  |  |  |  |
| Partial/subtotal gastrectomy | 0.39 | 0.32-0.47 | **< 0.001** |  | 0.50 | 0.34-0.74 | **0.001** |
| Total/near-total gastrectomy | 0.59 | 0.48-0.71 | **< 0.001** |  | 0.74 | 0.46-1.17 | 0.197 |
| Gastrectomy (NOS) | 0.72 | 0.48-1.07 | 0.104 |  | NE | NE | NE |
| **Chemotherapy, yes** | 0.51 | 0.43-0.59 | **< 0.001** |  | NA | NA | NA |
| **Radiotherapy, yes** | 0.60 | 0.49-0.73 | **< 0.001** |  | NA | NA | NA |

^1^Hazard ratios for associations of ≥ versus < 33 examined lymph nodes with overall survival were computed by Cox proportional hazards regression adjusting for year of diagnosis, sex, age, tumor location, local invasion, differentiation, size, metastatic lymph node number, and resection type. Statistically significant *P* values are shown in bold.

^2^Lesser curvature, greater curvature, overlapping lesion of stomach, and stomach (NOS).

Adj., adjusted; HR, hazard ratio; CI, confidence interval; NOS, not otherwise specified; NE, not estimable; NA, not available.

**Table S4.** Association of ≥ versus < 33 examined lymph nodes with lower-to-higher nodal stage migration in resected non-metastatic gastric adenocarcinoma patients with ≥ 1 examined lymph node using *multivariable*-adjusted multinomial logistic regression, overall and in *subgroups* by patient, tumor, and treatment factors^1^

| **Stratification** | **The US** | | |  | **China** | | |
| --- | --- | --- | --- | --- | --- | --- | --- |
|  | **OR** | **95% CI** | ***P_OR_*** |  | **OR** | **95% CI** | ***P_OR_*** |
| **Overall** | 2.21 | 1.95-2.52 | **< 0.001** |  | 1.69 | 1.29-2.21 | **< 0.001** |
| **Sex** |  |  |  |  |  |  |  |
| Male | 2.09 | 1.78-2.47 | **< 0.001** |  | 1.49 | 1.08-2.05 | **0.016** |
| Female | 2.42 | 1.97-2.99 | **< 0.001** |  | 2.40 | 1.43-4.03 | **0.001** |
| **Age group** |  |  |  |  |  |  |  |
| < 50 years | 2.23 | 1.59-3.12 | **< 0.001** |  | 1.83 | 1.08-3.10 | **0.024** |
| 50-59 years | 2.05 | 1.56-2.71 | **< 0.001** |  | 0.97 | 0.57-1.64 | 0.902 |
| 60-69 years | 2.17 | 1.70-2.77 | **< 0.001** |  | 2.88 | 1.75-4.74 | **< 0.001** |
| 70-79 years | 2.22 | 1.70-2.89 | **< 0.001** |  | 2.18 | 0.81-5.91 | 0.124 |
| ≥ 80 years | 2.92 | 1.96-4.35 | **< 0.001** |  | NE | NE | NE |
| **Tumor location** |  |  |  |  |  |  |  |
| Gastric cardia | 1.67 | 1.26-2.22 | **< 0.001** |  | 2.97 | 1.64-5.40 | **< 0.001** |
| Gastric fundus/body | 1.77 | 1.28-2.44 | **0.001** |  | 1.50 | 0.85-1.64 | 0.163 |
| Gastric antrum/pylorus | 3.11 | 2.42-4.00 | **< 0.001** |  | 1.52 | 1.05-2.22 | **0.028** |
| Other^2^ | 2.24 | 1.80-2.78 | **< 0.001** |  | NA | NA | NA |
| **Tumor local invasion** |  |  |  |  |  |  |  |
| Lamina propria/submucosa | 1.51 | 1.04-2.20 | **0.031** |  | 1.57 | 0.60-4.11 | 0.360 |
| Muscularis propria/subserosa | 2.06 | 1.74-2.45 | **< 0.001** |  | 1.28 | 0.63-2.62 | 0.497 |
| Serosa | 2.99 | 2.28-3.91 | **< 0.001** |  | 1.81 | 1.27-2.58 | **0.001** |
| Adjacent structures | 3.40 | 2.03-5.70 | **< 0.001** |  | 2.54 | 1.18-5.49 | **0.018** |
| **Differentiation** |  |  |  |  |  |  |  |
| Well | 0.70 | 0.22-2.25 | 0.551 |  | 3.30 | 0.98-11.19 | 0.055 |
| Intermediate | 1.87 | 1.42-2.46 | **< 0.001** |  | 1.00 | 0.60-1.67 | 0.987 |
| Poor/undifferentiated | 2.40 | 2.07-2.79 | **< 0.001** |  | 2.15 | 1.53-3.03 | **< 0.001** |
| **Tumor size group** |  |  |  |  |  |  |  |
| < 2 cm | 2.19 | 1.45-3.29 | **< 0.001** |  | 1.91 | 0.53-6.90 | 0.323 |
| 2-4 cm | 1.85 | 1.43-2.40 | **< 0.001** |  | 1.29 | 0.82-2.05 | 0.273 |
| 4-6 cm | 2.38 | 1.86-3.04 | **< 0.001** |  | 1.83 | 1.15-2.92 | **0.011** |
| 6-8 cm | 2.46 | 1.80-3.36 | **< 0.001** |  | 2.57 | 1.21-5.46 | **0.014** |
| ≥ 8 cm | 2.38 | 1.76-3.22 | **< 0.001** |  | 1.73 | 0.70-4.29 | 0.237 |
| **Resection type** |  |  |  |  |  |  |  |
| Partial/subtotal gastrectomy | 2.31 | 1.96-2.73 | **< 0.001** |  | 1.46 | 1.04-2.04 | **0.030** |
| Total/near-total gastrectomy | 2.14 | 1.70-2.68 | **< 0.001** |  | 2.33 | 1.44-3.78 | **0.001** |
| Gastrectomy (NOS) | 1.69 | 1.04-2.73 | **0.034** |  | NE | NE | NE |
| **Chemotherapy, yes** | 2.25 | 1.93-2.62 | **< 0.001** |  | NA | NA | NA |
| **Radiotherapy, yes** | 2.70 | 2.19-3.34 | **< 0.001** |  | NA | NA | NA |

^1^Odds ratios for association of examined lymph node number (≥ versus < 33) with serial advancement in nodal stage overall and in stratifications were computed using multivariable multinomial logistic regression models adjusting for year of diagnosis, sex, age, tumor location, local invasion, differentiation, size, and resection type. Statistically significant *P* values are shown in bold.

^2^Lesser curvature, greater curvature, overlapping lesion of stomach, and stomach (NOS).

Adj., adjusted; OR, odds ratio; CI, confidence interval; NOS, not otherwise specified; NE, not estimable; NA, not available.


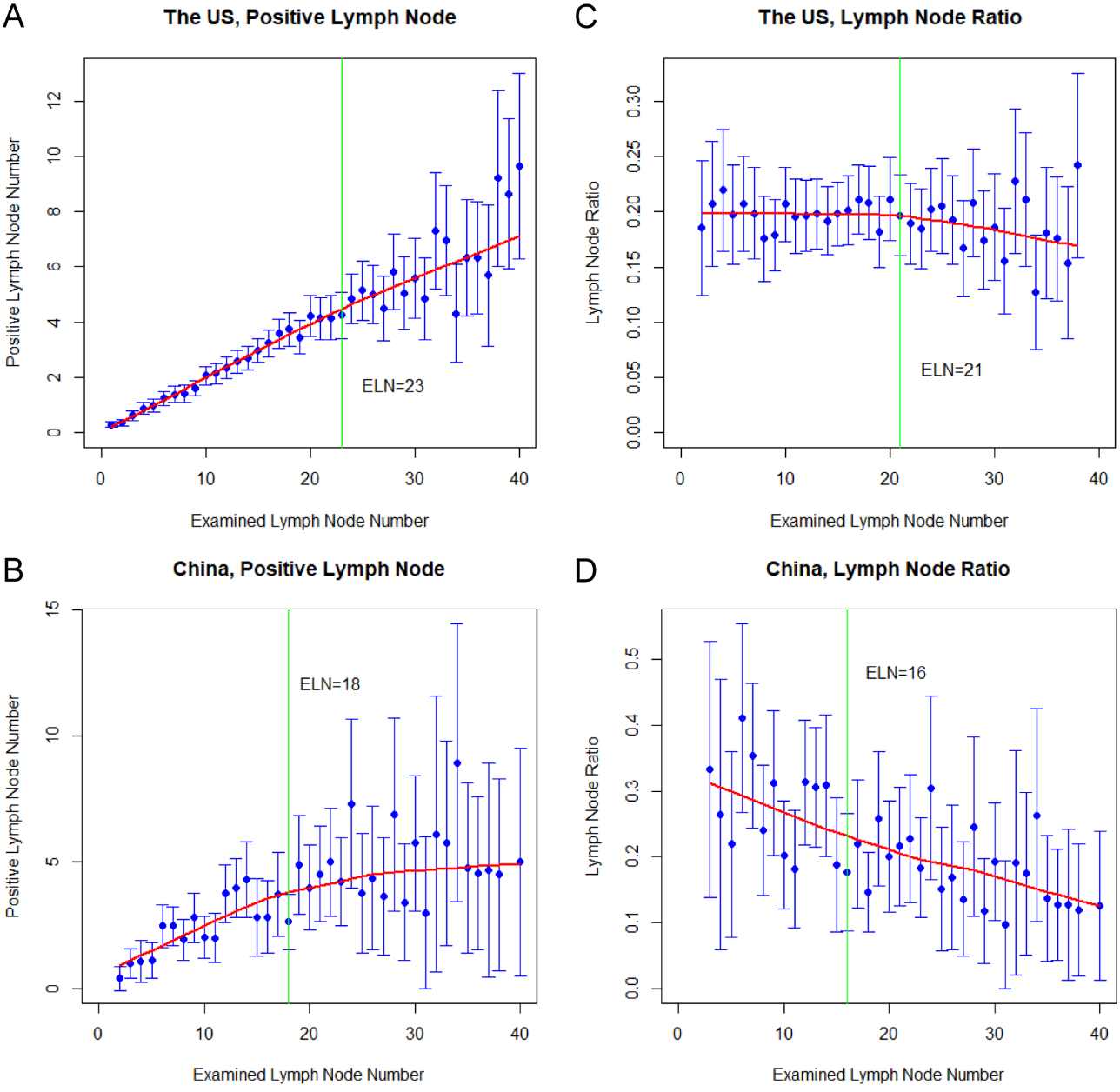


**Figure S1.** Associations of examined lymph node number (ELN) with positive lymph node number (**A** and **B**) and lymph node ratio (**C** and **D**) in the US and China cohorts. The mean estimates and corresponding 95% confidence intervals for the variables associated with ELN number are shown in blue. LOWESS smoother-fitted curves with a default fitting bandwidth of 2/3 are shown in red. The structural breakpoints determined using the Chow test for the variables are shown in green.
